# CML38 is involved in NO-induced inhibition of hypocotyl elongation in Arabidopsis

**DOI:** 10.3389/fpls.2025.1684245

**Published:** 2025-10-08

**Authors:** Dongsheng Wang, Zhaoyun Li, Xiaoduo Zhang, Yanjie Bian, Weizhong Liu

**Affiliations:** College of Life Science, Shanxi Normal University, Taiyuan, Shanxi, China

**Keywords:** nitric oxide, calcium, CML38, light signaling, phytohormone signaling

## Abstract

Calcium ions (Ca^2+^) are vital in plants, functioning both as structural cellular components and key secondary messengers that regulate growth, development, and stress responses. Nitric oxide (NO), a ubiquitous gaseous signaling molecule in organisms, also modulates diverse plant physiological processes. These two signaling molecules form a bidirectional interaction network, though the molecular mechanisms underlying their crosstalk remain poorly understood. Previous studies suggest that the calmodulin-like (CML) protein family mediates the interplay between NO and Ca^2+^ signaling. Our earlier RNA-seq data indicated that *CML38* expression is responsive to exogenous NO in Arabidopsis seedlings, prompting the hypothesis that NO and Ca^2+^ signaling may interact with each other via CML38 regulation. To test this hypothesis, we employed *Arabidopsis thaliana* as a model plant and integrated genetic, biochemical, and molecular approaches to elucidate CML38’s role in NO-mediated hypocotyl growth inhibition. Our findings demonstrate that NO treatment significantly suppresses hypocotyl elongation in wild-type plants but not in CML38 loss-of-function mutant. CML38 binds Ca^2+^ and its calcium-binding capacity is unaffected by NO. Transcriptomic analysis revealed that CML38 participates in the crosstalk between NO and Ca^2+^ signaling, light signaling, as well as phytohormones. This study advances our understanding of the NO-Ca^2+^ interaction network in plants and provides insights into the molecular mechanisms by which these signals coordinately regulate plant growth and stress adaptation.

## 1 Introduction

Calcium ions (Ca^2+^) act as universal secondary messengers in plants, coordinating growth, development, and stress responses through dynamic regulation of cytosolic Ca^2+^ concentration ([Ca^2+^]cyt) (Kudla et al., 2010). Under homeostatic conditions, [Ca^2+^]cyt is maintained at nanomolar levels, but plants rapidly generate stimulus-specific Ca^2+^ signatures in response to environmental cues (e.g., light, temperature), hormonal pathways, pathogen challenges, and abiotic stresses (e.g., salinity, drought). The [Ca^2+^]cyt fluctuations trigger downstream signaling cascades that regulate distinct physiological responses (Pirayesh et al., 2021). Importantly, Ca^2+^ signals extensively interact with other secondary messengers, including nitric oxide (NO). NO modulates Ca^2+^ dynamics through activation of Ca^2+^-dependent protein kinases (e.g., CDPKs) and post-translational modification of Ca^2+^ channels via *S*-nitrosylation (Courtois et al., 2008; Jeandroz et al., 2013; Hu et al., 2022). This intricate crosstalk between NO and Ca^2+^ signaling enables precise spatiotemporal regulation of cellular processes, ranging from cell division and elongation to complex physiological adaptations, forming an integrated signaling network that processes multiple environmental and developmental cues.

Ca^2+^ response proteins decode cytosolic Ca^2+^ fluctuations into specific physiological outputs, with calmodulin-like proteins (CMLs) representing a large family of Ca^2+^ sensors with specialized functions beyond the highly conserved calmodulin (CaM). Arabidopsis CMLs possess the characteristic EF-hand Ca^2+^-binding domains and exhibit remarkable functional diversification in plant growth and stress responses (McCormack and Braam, 2003). During early development, CMLs precisely regulate seed germination through hormone signaling pathways. CML14 and CML24 promote germination, whereas CML39 acts as a negative regulator by enhancing ABA sensitivity and suppressing gibberellin responses, with *cml39* showing accelerated germination and reproductive defects including shortened siliques and reduced seed set (Delk et al., 2005; Bender et al., 2013; Symonds et al., 2024). In reproductive processes, multiple CMLs (CML3, 14, 16, 49) show pollen-specific expression patterns, with CML24 and CML25 directly facilitating pollen tube growth via modulation of potassium channel activity, and CML36 participating in floral transition through activation of ACA8 Ca^2+^-ATPase (Tsai et al., 2007; Wang et al., 2008, 2015; Yang et al., 2014). CMLs also integrate Ca^2+^ signaling with developmental senescence and environmental responses. MdCML15 accelerates ethylene-mediated leaf and fruit senescence in apple through formation of a transcriptional regulatory complex with MdVQ10 and MdWRKY75, while papaya CpCML15 directly promotes ripening by modulating senescence-related gene networks (Ding et al., 2018). In light signaling, CML39 functions as a photoreceptor-coupled Ca^2+^ sensor regulating photomorphogenesis, with mutants displaying aberrant hypocotyl elongation (Bender et al., 2013), whereas CML9 mediates root growth responses to microbial elicitors like flagellin (Leba et al., 2012). The functional diversification of CMLs enables plants to generate appropriate responses to myriad internal and external stimuli through Ca^2+^ signaling networks.

Based on our previous transcriptomic data, which showed that NO treatment markedly alters the expression of several CML family members, we focused on the five most responsive genes: *CML23*, *CML24*, *CML37*, *CML38*, and *CML39* (Ren et al., 2024). Among these, phenotypic analysis of loss-of-function mutants revealed that both *cml23* and *cml38* mutants were insensitive to NO-mediated inhibition of hypocotyl elongation (data unpublished). Since the interaction between NO and CML23 has been extensively detailed elsewhere (Wang et al., 2025), we focused this study on characterizing the novel role of CML38 in NO signaling pathway. CML38 is a multifunctional Ca^2+^ response protein, which integrates diverse signaling inputs—including Ca^2+^ fluctuations, hormonal (e.g., brassinosteroid) cues, nutrient (e.g., nitrate) availability, and environmental (e.g., hypoxia) stimuli—to orchestrate plant development and stress adaptation (Lokdarshi et al., 2016; Field et al., 2021; Song et al., 2021). It exerts its regulatory roles through modulating gene expression, mRNA metabolism, and autophagy, positioning it as a central hub in cellular signaling networks.

Previous studies have shown that the CML protein family plays a role in the crosstalk between NO and Ca^2+^ signaling, potentially by translating cytoplasmic Ca²^+^ fluctuations into cellular responses via modulation of NO biosynthesis or turnover (Tsai et al., 2007). Here, we investigate the molecular mechanisms by which CML38 mediates NO-induced suppression of hypocotyl elongation, providing direct evidence that CML38 operates downstream of NO perception to regulate Ca^2+^-dependent signaling pathways.

## 2 Materials and methods

### 2.1 Plant growth condition

*Arabidopsis thaliana* ecotype Columbia-0 (Col-0) was used as the wild-type. The T-DNA insertion mutant *cml38* (SALK_066538C) was obtained from the Arashare Center (Fuzhou, China) and genotypically verified by PCR (**Supplementary Figure S1**).

Seeds were surface-sterilized sequentially with 70% (v/v) ethanol for 30 s, followed by 10% (v/v) sodium hypochlorite for 15min, and rinsed five times with sterile distilled water. Sterilized seeds were stratified at 4°C for 48h to synchronize germination, then sown vertically on half-strength Murashige and Skoog (1/2 MS) medium in growth chambers maintained at 22°C under a 12-h photoperiod. After 48h of germination, uniform seedlings were transferred to fresh 1/2 MS plates (9cm diameter) containing the following treatments: sodium nitroprusside (SNP; NO donor) fumigation, 1 mM CaCl_2_, 1 mM EGTA (calcium chelator), or 50 μM ruthenium red (RR; calcium channel inhibitor). For SNP fumigation, sterilized microcentrifuge tubes were placed on the medium surface, and 10 μL of 100 mM SNP solution was added to each tube to allow gradual NO release under light conditions. The Petri dishes were sealed and the SNP solution was applied only once at the beginning of the experiment. Seedlings were grown for 7 additional days under the same conditions. Hypocotyl length was quantified using ImageJ software (NIH, USA) for precise image analysis and morphometric measurements. Graphs were generated using GraphPad Prism 9 (GraphPad Software, USA). Statistical significance between two groups was determined using unpaired two-tailed Student’s t-test.

### 2.2 Bioinformatics analysis

The Ca^2+^ binding sites of CML38 were predicted using the MIB2 online server (Lu et al., 2022), and the highest-scoring model was visualized in PyMOL (version 2.4.0).

### 2.3 Recombinant CML38 protein expression and purification

*Arabidopsis thaliana* ecotype Columbia-0 (Col-0) was used as the wild-type. The T-DNA inser The Arabidopsis *CML38* gene was synthesized and cloned into the EcoRI and SalI restriction sites of the pET28a(+) prokaryotic expression vector (GenScript Biotech Corporation, Nanjing, China), and verified by sequencing. The recombinant pET28a: CML38 plasmid was then transformed into *E. coli* BL21 (DE3) competent cells. Transformed colonies were cultured in LB medium at 37°C to OD_600_=0.6, then induced with 0.2 mM isopropyl β-D-1-thiogalactopyranoside (IPTG) at 16°C for 18h. His-tagged recombinant CML38 protein was subsequently purified using BeyoGold™ His-tag Purification Resin (Beyotime, P2218) according to the manufacturer’s protocol.

### 2.4 [Ca^2+^]cyt concentration detection

The Fluo-4 AM fluorescence probe (Beyotime) was used to detect [Ca^2+^]cyt concentration in Arabidopsis hypocotyls, and the detection process was carried out on a BX53 fluorescence microscope (Olympus, Tokyo, Japan). Hypocotyl samples from 9-day-old seedlings were first incubated to load Fluo-4 AM probe, following the manufacture’s protocol. Then the samples were treated with 20 μM GSNO (an alternative NO donor) for 10 minutes, as this donor was chosen to avoid light exposure—which is detrimental to the Fluo-4 AM probe yet essential for NO release from SNP. The relative fluorescence intensity (indicative of [Ca^2+^]cyt concentration) was quantified using ImageJ. The graphing and statistical analysis methods were the same as those employed in the phenotypic detection assay.

### 2.5 Ca^2+^-binding assay

Electrophoresis mobility shift assays were performed as described (Vanderbeld and Snedden, 2007) with modifications. Prior to denaturation, purified recombinant protein was treated with either 200 µM fresh SNP solution, or 48h light-exposed SNP (NO-depleted control). The denatured protein samples were then incubated with 5 mM CaCl_2_ or 5 mM EGTA for 30min at 4°C, then separated by SDS-PAGE using gels and running buffers containing either 2 mM CaCl_2_ or 2 mM EGTA to maintain consistent Ca^2+^ conditions.

### 2.6 RNA extraction and RT-qPCR analysis

The aboveground parts of 9-day-old seedlings were flash-frozen in liquid nitrogen and homogenized. Total RNA was isolated using CTAB-PBIOZOL reagent (Takara, Beijing, China) following the manufacturer’s protocol, and integrity was assessed with an Agilent 2100 Bioanalyzer.

Quantitative real-time PCR (RT-qPCR) analysis was applied using the QuantStudio™ 5 Real-Time PCR System (Applied Biosystems) in 96-well plates. Each 20 µL reaction contained 10 µL 2× PerfectStart™ Green qPCR SuperMix (Transgene, Beijing, China), 0.5 µL each of forward and reverse primers (listed in **Supplementary Table S1**) (10 µM), 1 µL cDNA template (~20 ng), and 8 µL nuclease-free water. Thermal cycling conditions: initial denaturation at 94°C for 3min, followed by 40 cycles of 94°C for 30 s, 60°C for 15 s, and 94°C for 10 s. Gene expression levels were normalized to the reference gene *β-ACTIN* using the ΔΔCt method, with technical triplicates performed for each sample to ensure data reproducibility, and melting curve analysis was conducted to verify amplification specificity.

### 2.7 Sequencing

Libraries were constructed as follows: (1) genomic DNA elimination and mRNA enrichment using oligo(dT) magnetic beads; (2) RNA fragmentation in NEBNext First Strand Synthesis Buffer (5×) with divalent cations at 94°C for 5–7 minutes; (3) double-stranded cDNA synthesis using M-MuLV Reverse Transcriptase (RNase H-) and DNA Polymerase I; (4) cDNA end repair, 3’ adenylation, and NEBNext adaptor ligation; and (5) size selection (250–300 bp fragments) with AMPure XP beads (Beckman Coulter, Germany).

The libraries underwent: (i) USER Enzyme (NEB) treatment (37°C, 15min; 95°C, 5min); (ii) 12-cycle PCR amplification with Phusion High-Fidelity DNA polymerase; and (iii) final purification with AMPure XP beads. Quality control was performed using the Agilent Bioanalyzer 2100 system prior to 2 × 150 bp paired-end sequencing on the Illumina NovaSeq 6000 platform (Wekemo Tech Group, Shenzhen), generating approximately 40 million reads per sample.

### 2.8 Analysis of deferentially expressed genes

RNA-seq analysis generated robust datasets with each biological replicate producing >46 million raw paired-end reads. Following sequencing, we implemented a rigorous quality control pipeline: (1) adapter trimming and quality filtering using FastQC (v0.23.1) with custom Perl scripts to remove low-quality bases (Q<20), poly-N sequences (>10% N content), and reads shorter than 50 bp; (2) alignment of processed reads to the Arabidopsis reference genome (GCF_000001735.4_TAIR10.1) using HISAT2 (v2.0.5) with default parameters, achieving >85% mapping efficiency across all samples; and (3) transcript quantification through reference-guided assembly with StringTie (v1.3.3b), expressing gene abundance as FPKM values after normalization.

For differential expression analysis, we employed DESeq2 (v1.4.5) with a stringent threshold of |log2FC| > 1 (equivalent to 2-fold change) and P-value < 0.05. Functional annotation of differentially expressed genes (DEGs) involved: (i) GO term enrichment analysis using GOSeq (v1.34.1) with Wallenius approximation to correct for transcript length bias; and (ii) pathway mapping to KEGG databases through the ClusterProfiler R package (v3.14.3), with significance determined by FDR < 0.05 after Benjamini-Hochberg correction. All analyses were performed with triplicate biological samples.

## 3 Results

### 3.1 CML38 is involved in NO inhibition of hypocotyl elongation

**Figure 1:**
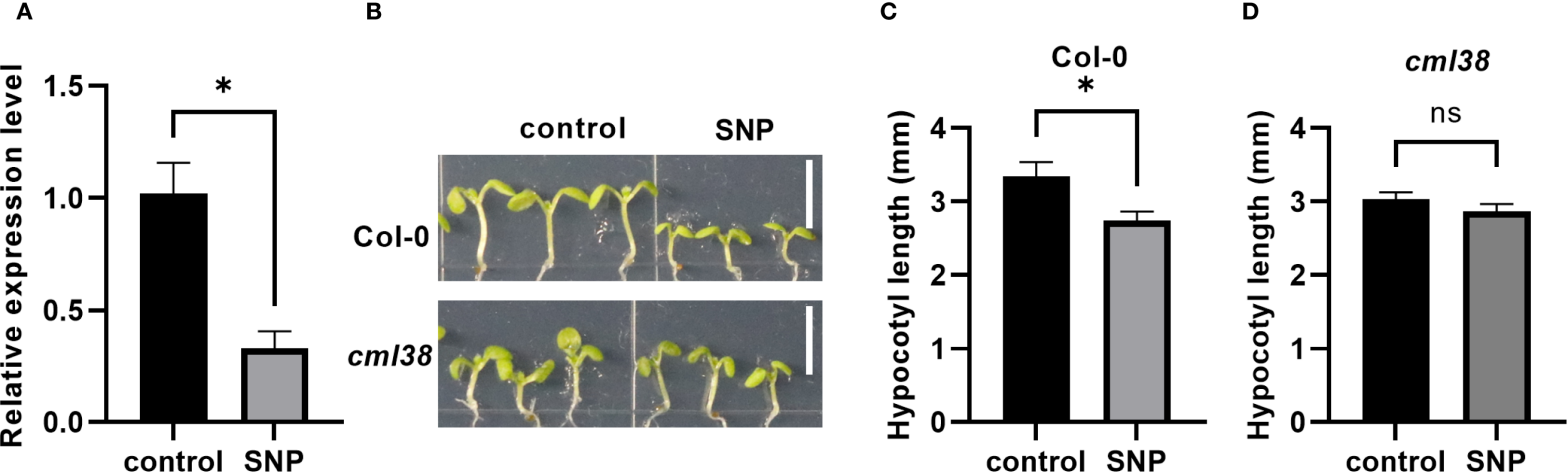
CML38 mediates NO-induced inhibition of hypocotyl elongation. **(A)** *CML38* expression level in Col-0 under NO treatment. **(B)** Phenotype of Col-0 and *cml38*. Scale bars represent 3mm. **(C)** Hypocotyl length of Col-0. **(D)** Hypocotyl length of *cml38*. Data are presented as mean ± SEM; *p < 0.05, ns, not significant.

*CML38* was previously identified as a NO-responsive gene in Arabidopsis seedling (Ren et al., 2024). Here, RT-qPCR showed that 7-day exogenous NO treatment significantly downregulated *CML38* expression in Col-0 (p < 0.05; **Figures 1A, B**). Phenotypic analysis revealed that while NO effectively inhibited hypocotyl growth in Col-0 (p < 0.05), but not in *cml38* (**Figures 1C, D**). These results demonstrate that CML38 functions as a key mediator in NO-induced suppression of hypocotyl elongation.

### 3.2 Ca^2+^ modulates the regulation of NO on CML38

**Figure 2:**
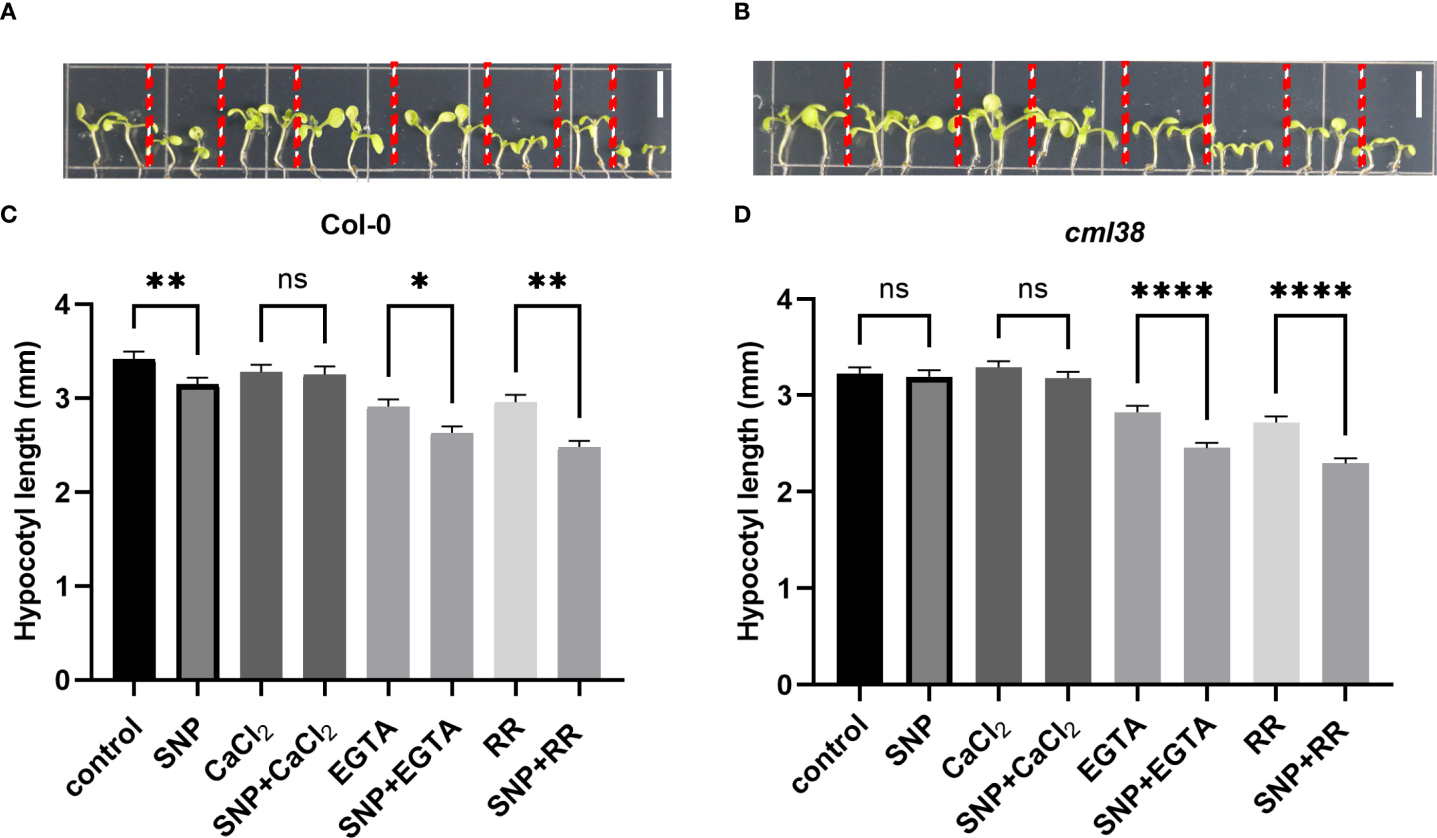
Ca^2+^ homeostasis modulates NO signaling in hypocotyl elongation. **(A)** Phenotype of Col-0. **(B)** Phenotype of *cml38*. **(C)** Hypocotyl length of Col-0. **(D)** Hypocotyl length of *cml38*. Scale bars in A and B represent 3mm. Data are presented as mean ± SEM; *p < 0.05, **p < 0.01, ****p < 0.0001, ns, not significant.

**Figure 3:**
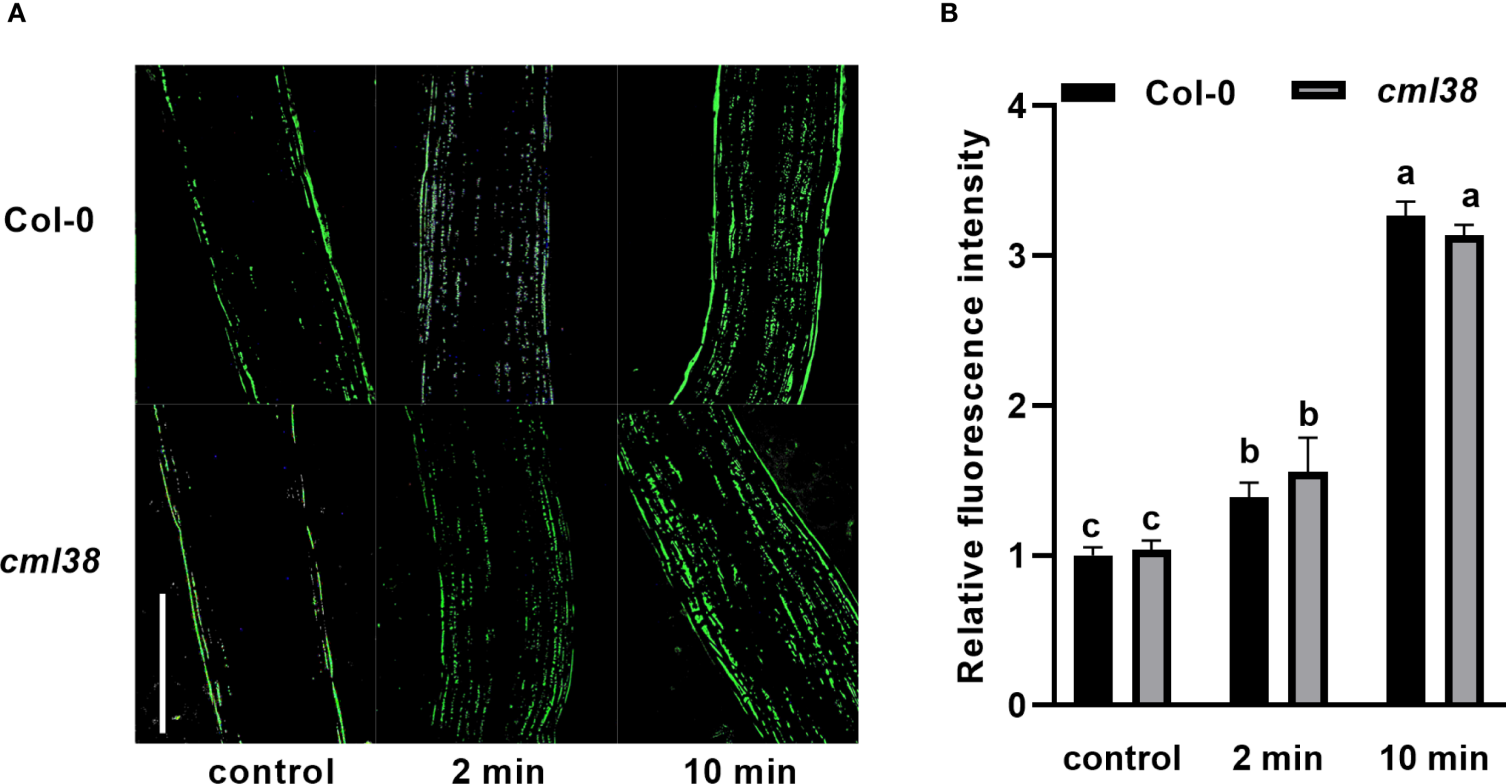
[Ca^2+^]cyt elevation in response to GSNO treatment is independent of CML38. **(A)** Representative fluorescent images of hypocotyls from Col-0 and *cml38* treated with 20 μM GSNO for the indicated times. Scale bars represent 200 µm. **(B)** Quantification of relative fluorescence intensity from **(A)**. Different letters indicate significant differences (p < 0.05).

To investigate whether the regulatory effect of NO on CML38 is Ca^2+^-dependent, we examined hypocotyl elongation under pharmacological modulation of Ca^2+^ availability (**Figure 2**). Exogenous CaCl_2_ partially alleviated NO-induced hypocotyl growth inhibition in Col-0. Conversely, disrupting Ca^2+^ signaling with either the extracellular chelator EGTA or the channel blocker RR enhanced NO sensitivity in *cml38*, suggesting CML38 is involved in NO-Ca^2+^ crosstalk. However, *cml38* mutants exhibited a similar trend to Col-0 in NO-triggered [Ca^2+^]cyt increases (**Figure 3**), indicating that CML38 may act downstream of the initial calcium flux to mediate the growth response.

### 3.3 NO does not affect CML38’s Ca^2+^-binding capability

**Figure 4:**
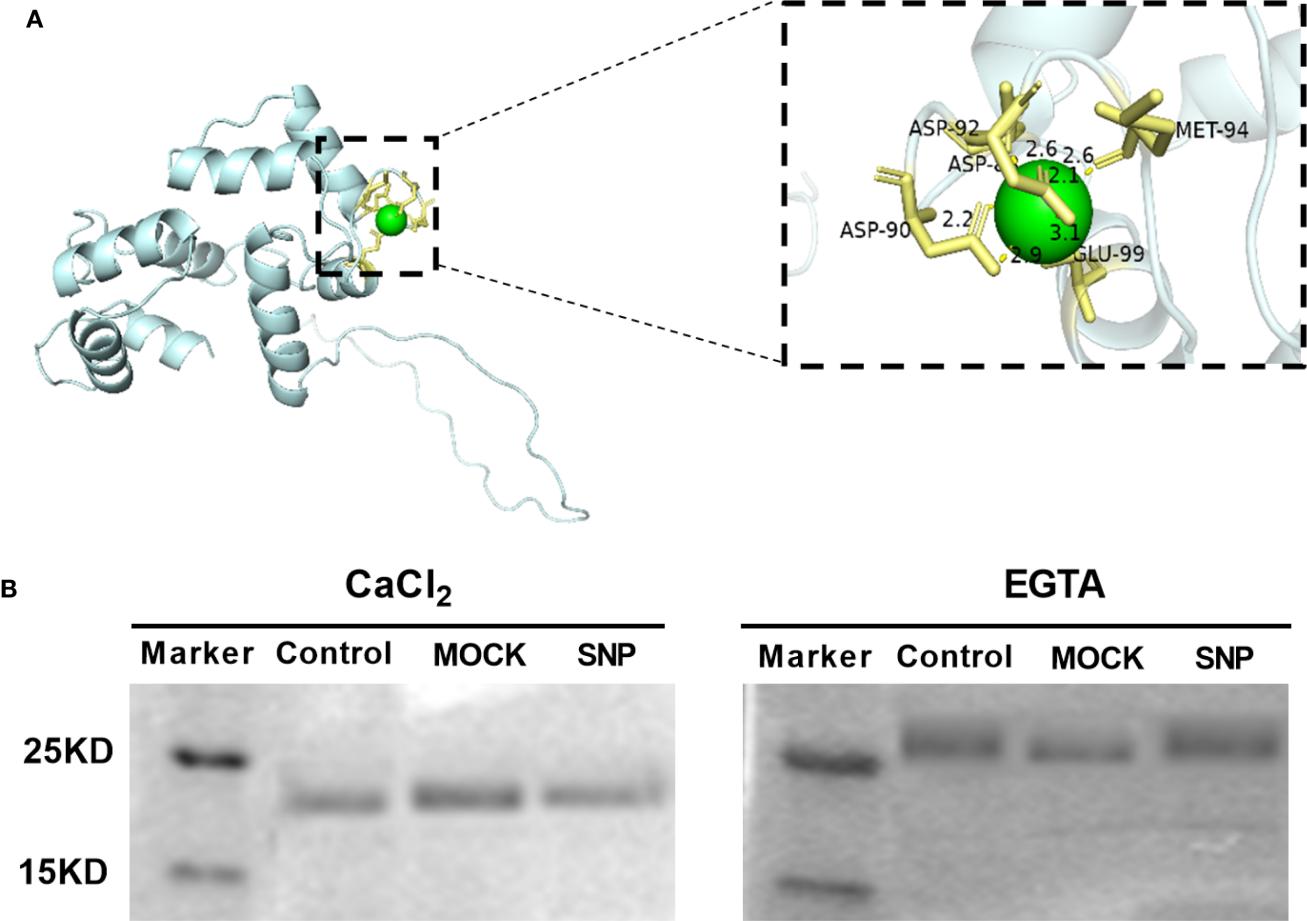
CML38 maintains Ca^2+^-binding activity independent of NO signaling. **(A)** Structural prediction of conserved Ca^2+^-binding site in CML38. **(B)** Ca^2+^-dependent electrophoretic mobility shift assay of recombinant CML38 under different treatments: Experimental: 200 µM fresh SNP (NO-producing); Negative control: equal volume of distilled H_2_O; Mock control: 200 µM light-exposed SNP (48-hour irradiated, NO-depleted).

The CML protein family represents a group of putative Ca^2+^ sensors in plants. CML38 is a 177-amino acid protein with a predicted molecular mass of 23 kDa. Notably, CML38 contains three evolutionarily conserved EF-hand motifs, characteristic calcium-binding domains that suggest its functional role in calcium signal perception and transduction. To assess the Ca^2+^-binding capability of CML38, we performed structural predictions. Computational modeling revealed that CML38 likely coordinates Ca^2+^ ions through specific residues including Met, Asp and Glu (**Figure 4A**).

SDS-PAGE mobility shift assays confirmed Ca^2+^-binding by CML38: faster migration was observed in the presence of Ca^2+^ versus EGTA (**Figure 3B**). To test NO effects, CML38 was treated with fresh SNP (NO-releasing) or light-exposed SNP (NO-depleted control). NO did not alter CML38’s migration pattern under identical Ca^2+^ conditions, indicating NO does not affect its Ca^2+^ binding capacity (**Figure 4B**).

### 3.4 Genome-wide identification of CML38-target genes in NO signaling

**Figure 5:**
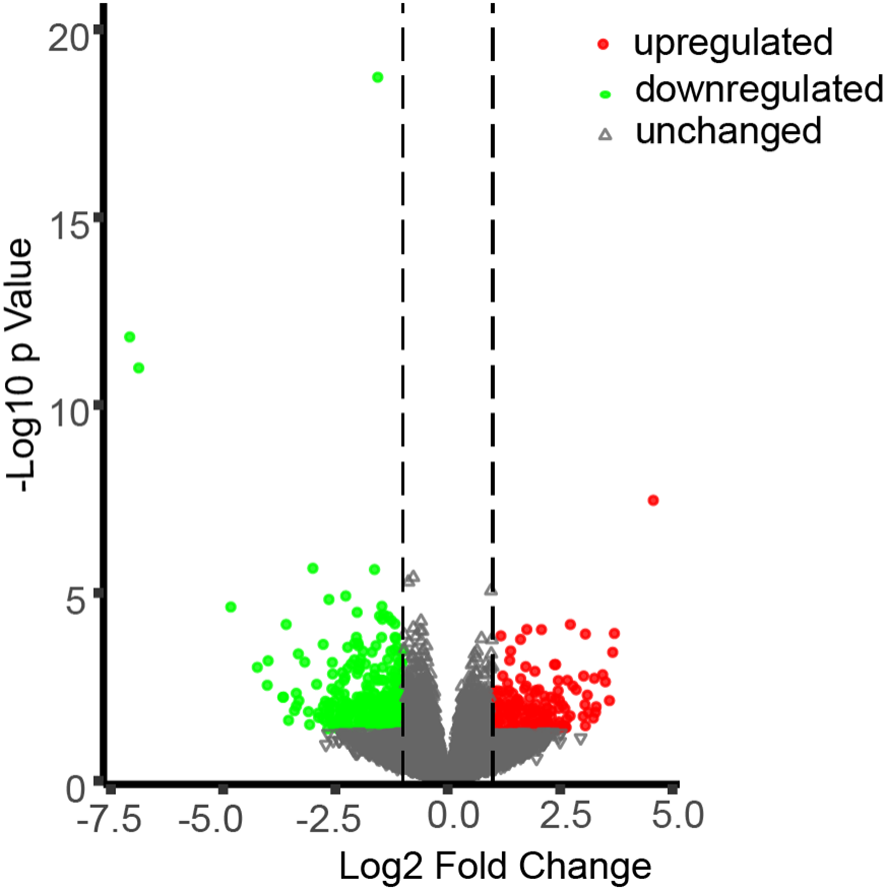
Differentially expressed genes (DEGs) in *cml38* under SNP-treated condition. Green, red and grey symbols represent for significantly down-regulated, upregulated and unchanged genes in *cml38*, compared to Col-0 (|log2FC| > 1, p < 0.05).

To gain deeper insights into the molecular pathways through which CML38 mediates NO-dependent regulation of hypocotyl elongation, we conducted comparative transcriptomic analysis between Col-0 and *cml38* under SNP-treated condition. Using stringent thresholds (|log2FC| > 1, p < 0.05), we found that CML38 deficiency significantly altered the Arabidopsis transcriptome, with 168 genes significantly up-regulated and 351 genes down-regulated (**Figure 5**).

**Figure 6:**
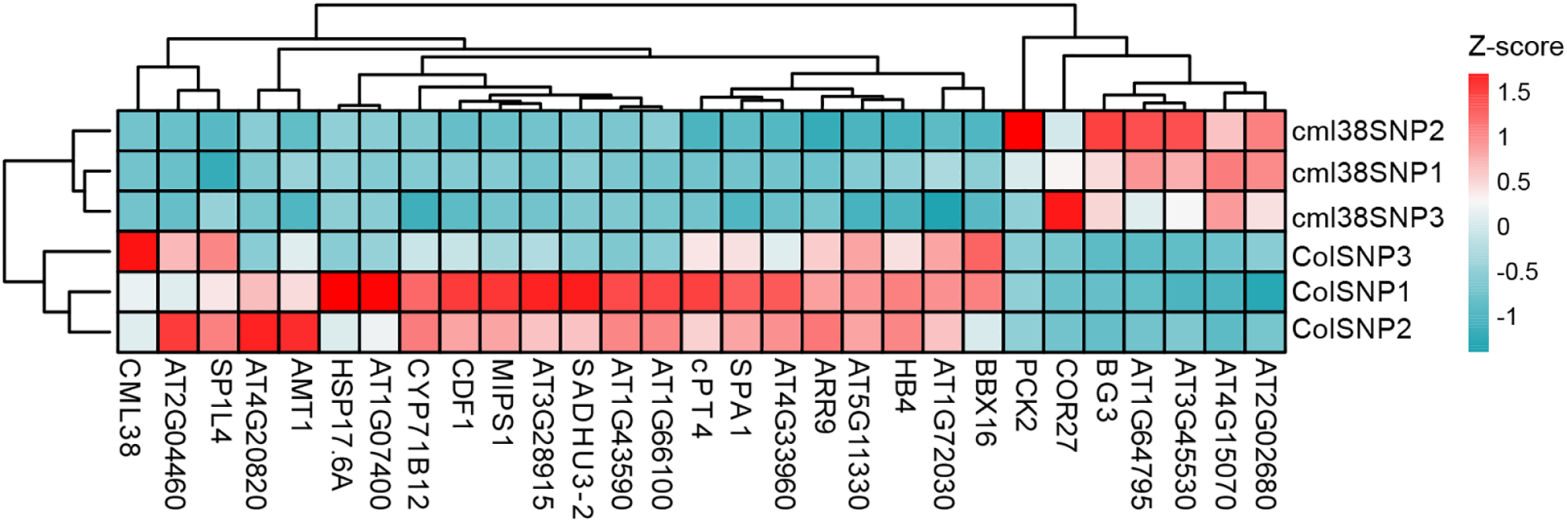
Unsupervised hierarchical clustering of 30 DEGs across all samples. The heatmap displays z-score normalized expression across all samples. The color key indicates relative expression levels: red represents higher expression, and blue represents lower expression.

To visually summarize the transcriptomic landscape and validate the distinctiveness of the experimental groups, we performed unsupervised hierarchical clustering of the samples based on the expression profiles of the top 30 most DEGs between Col-0 and *cml38*. As shown in **Figure 6**, the resulting heatmap revealed a clear and striking separation of the samples into two primary clusters that corresponded perfectly to Col-0 and *cml38*. This pronounced segregation provides strong evidence that the transcriptional profiles are fundamentally distinct between the two lines.

### 3.5 GO enrichment analysis of CML38-regulated DEGs under NO treatment

GO enrichment analysis identified a total of 769 GO terms, reflecting the broad regulatory scope of CML38 in NO signaling. Considering light and phytohormones also play vital roles during hypocotyl elongation, we focused on three functionally relevant categories of GO terms: (1) calcium ion binding, signaling and homeostasis, (2) photoreceptor pathways (encompassing responses to red, far-red, and blue light responses), and (3) phytohormones biosynthesis and response. This allowed us to identify key regulatory networks potentially involved in CML38-dependent growth regulation.

Compared to Col-0, the *cml38* mutant exhibited 6 DEGs associated with Ca^2+^ binding, including 2 up-regulated genes (*NDB4* and AT4G13440) and 4 down-regulated genes (*CML38*, *CPK15*, *NIG1* and AT2G34030) genes in *cml38* (**Table 1**). Furthermore, *cml38* showed significant enrichment of 2 DEGs directly involved in Ca^2+^ transport and signal transduction: *OSCA2.1* and *GLUR2*. These results suggest that CML38 acts as a key regulator of NO-induced Ca^2+^ signaling, likely by modulating the expression of above-mentioned genes.

**Table 1:** Differential expressed genes associated with Ca^2+^ binding and signaling.

Gene ID	Gene name	Description	Change
AT1G76650	*CML38*	Calmodulin-like 38	down
AT2G20800	*NDB4*	NAD(P)H dehydrogenase B4	up
AT2G34030		Calcium-binding EF-hand family protein	down
AT4G13440		Calcium-binding EF-hand family protein	up
AT4G21940	*CPK15*	calcium-dependent protein kinase 15	down
AT5G46830	*NIG1*	calcium-binding transcription factor NIG1	down
AT1G58520	*OSCA2.1*	hypo-osmosensitive Ca^2+^-permeable channels	down
AT4G35290	*GLUR2*	glutamate receptor 2	down

Light is a primary environmental cue that tightly regulates hypocotyl elongation through distinct photoreceptor systems: red light inhibits elongation via phytochrome activation, far-red light reverses this inhibition to promote growth, and blue light independently suppresses elongation through cryptochromes and phototropins (Chen et al., 2023). Emerging evidence indicates that NO serves as a key modulator of these light-mediated growth responses (Sanz et al., 2015), but the molecular links between NO and light signaling remain poorly understood. To investigate whether CML38 contributes to NO-light signal integration, we analyzed the expression of light-responsive genes in Col-0 and cml38 under SNP treatment. Our transcriptomic data revealed striking differences in light-responsive gene expression profiles between the two genotypes. Specifically, CML38 deficiency altered the expression of 23 red/far-red light-responsive genes and 8 blue light-responsive genes in the presence of NO (**Tables 2**, **3**). These findings strongly suggest that CML38 participates in the integration of NO and light signaling pathways during hypocotyl development.

**Table 2:** Differential expressed genes related to red and far-red light.

Gene ID	Gene name	Description	Change
AT1G15550	*GA3OX1*	gibberellin 3-oxidase 1	down
AT1G58520	*OSCA2.1*	hypo-osmosensitive Ca^2+^-permeable channels	down
AT1G72030		Acyl-CoA N-acyltransferases (NAT) superfamily protein	down
AT2G40460		Major facilitator superfamily protein	down
AT3G05936		hypothetical protein	down
AT3G14810	*MSL5*	mechanosensitive channel of small conductance-like 5	down
AT3G21670		Major facilitator superfamily protein	down
AT5G02230		Haloacid dehalogenase-like hydrolase (HAD) superfamily protein	down
AT5G15600	*SP1L4*	SPIRAL1-like4	down
AT5G19970		GRAS family transcription factor family protein	down
AT5G54585		hypothetical protein	down
AT5G65890	*ACR1*	ACT domain repeat 1	down
AT2G05100	*LHCB2.1*	photosystem II light harvesting complex protein 2.1	down
AT2G44940		Integrase-type DNA-binding superfamily protein	down
AT2G46340	*SPA1*	SPA (suppressor of phyA-105) protein family	down
AT3G27690	*LHCB2.3*	photosystem II light harvesting complex protein 2.3	down
AT4G15233	*ABCG42*	ABC-2 and Plant PDR ABC-type transporter family protein	up
AT4G33980		hypothetical protein	up
AT5G24120	*SIGE*	sigma factor E	down
AT5G42900	*COR27*	cold regulated protein 27	up
AT2G44910	*HB4*	homeobox-leucine zipper protein 4	down
AT2G24540	*AFR*	Galactose oxidase/kelch repeat superfamily protein	down
AT2G46790	*PRR9*	pseudo-response regulator 9	down

**Table 3:** Differential expressed genes related to blue light.

Gene ID	Gene name	Description	Change
AT1G01520	*ASG4*	Homeodomain-like superfamily protein	down
AT2G46340	*SPA1*	SPA (suppressor of phyA-105) protein family	down
AT4G24700		hypothetical protein	down
AT4G33980		hypothetical protein	up
AT5G24120	*SIGE*	sigma factor E	down
AT5G26200		Mitochondrial substrate carrier family protein	down
AT5G42900	*COR27*	cold regulated protein 27	up
AT5G43630	*TZP*	zinc knuckle (CCHC-type) family protein	down

NO coordinates with multiple phytohormone pathways to regulate hypocotyl elongation. CML38 deficiency influenced NO-induced signal transduction of phytohormones. Our GO enrichment analysis revealed that CML38 deficiency significantly impacts NO-induced transcriptional changes in genes associated with 8 major phytohormone pathways: jasmonic acid, auxin, abscisic acid, brassinosteroid, salicylic acid, cytokinin, gibberellin and ethylene, were identified in SNP treated *cml38* (**Supplementary Table S2**).

### 3.6 KEGG enrichment analysis of DEGs

KEGG pathway enrichment analysis was performed to identify the metabolic pathways regulated by DEGs, elucidating how NO mediated by CML38 influences hypocotyl elongation in Arabidopsis. The results revealed that CML38 deficiency, in the presence of NO, led to the enrichment of 59 pathways, among which plant hormone signal transduction, circadian rhythm, MAPK signaling pathway, ubiquitin mediated proteolysis, zeatin, phenylpropanoid, cutin, suberine and wax biosynthesis, as well as alpha-linolenic acid, inositol phosphate and glycerophospholipid metabolism may involve in hypocotyl elongation (**Supplementary Table S3**).

### 3.7 Validation of transcriptome trofiles by RT-qPCR

**Figure 7:**
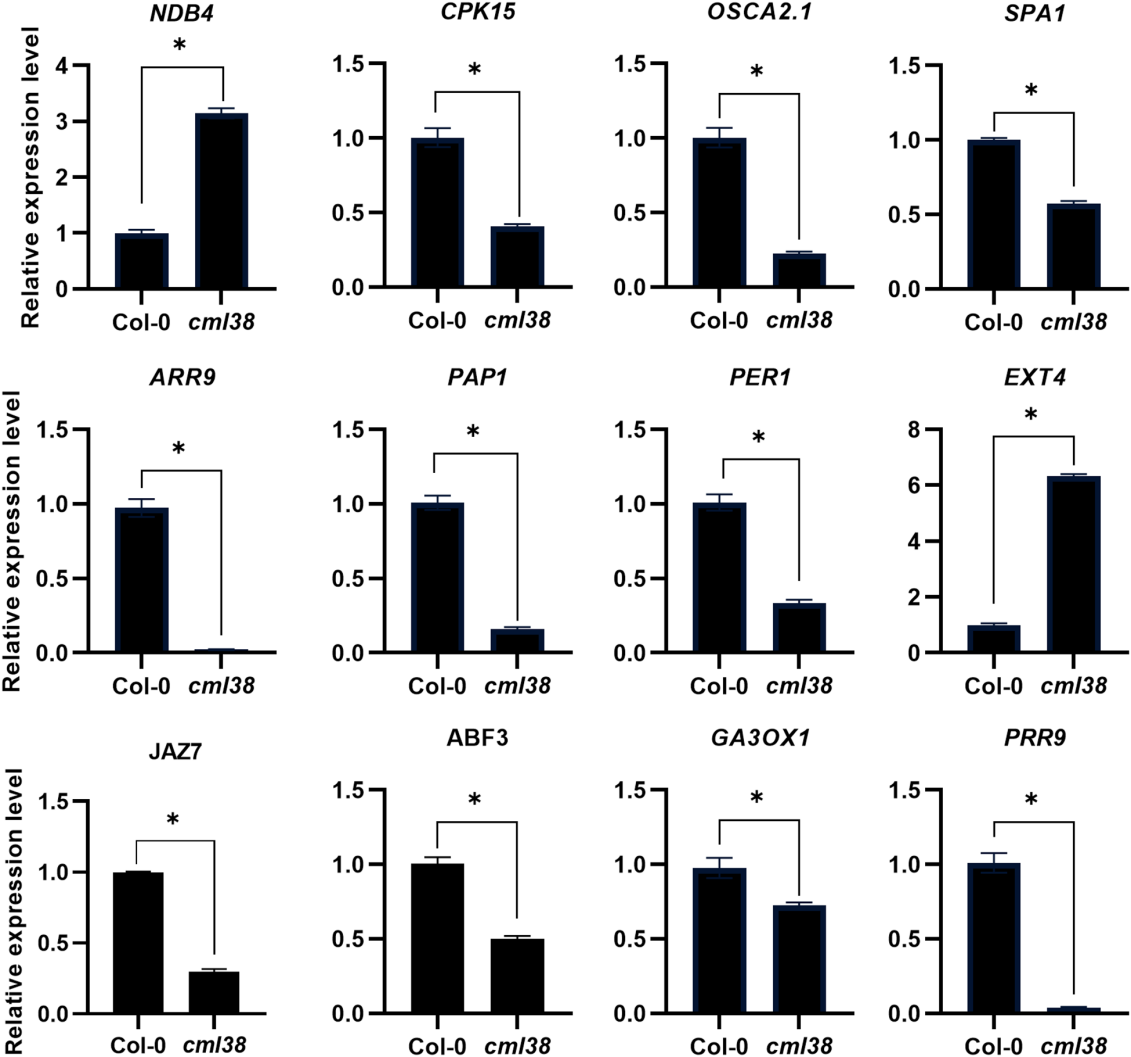
Validation of RNA-seq results and expression analysis of key genes related to Ca^2+^, light and phytohormone signaling by RT-qPCR. Data are presented as mean ± SEM; *p < 0.05.

To independently validate the reliability of the transcriptome profiles obtained by RNA-Seq, we performed reverse RT-qPCR on a subset of 12 DEGs. These genes were randomly selected from those implicated in the Ca^2+^ signaling, light signaling and phytohormone pathways, which our analysis suggested were interconnected with CML38-mediated signaling (**Tables 1**–**3**; **Supplementary Table S2**). The expression trends of all 12 genes as determined by RT-qPCR were entirely consistent with the patterns observed in the RNA-Seq data (**Figure 7**). This high degree of concordance demonstrates the high reproducibility and accuracy of our transcriptomic analysis and confirms the differential expression of key genes involved in the interplay between NO/Ca^2+^, light, and phytohormone signaling.

## 4 Discussion

Hypocotyl elongation represents a spatiotemporally regulated developmental process crucial for plant growth and development, playing a pivotal role in ensuring successful life cycle completion. This complex process is precisely controlled by both environmental factors (including light and temperature) and endogenous signaling molecules/growth regulators (Oh et al., 2014). Although exogenous NO treatment has been shown to inhibit hypocotyl elongation in Arabidopsis (Sanz et al., 2015), the precise molecular mechanisms underlying this regulation remain poorly understood. Among CMLs, current research has predominantly investigated their functions in seed germination, flowering, and fruit development (Munir et al., 2016; Wu et al., 2017). To date, two family members, CML23 and CML39, were demonstrated to participate in hypocotyl elongation regulation (Bender et al., 2013; Wang et al., 2025). Similar to CML23, our study identifies CML38 also as a crucial signaling hub integrating NO, Ca^2+^, light and phytohormones pathways to regulate hypocotyl elongation. However, mechanistic exploration reveals distinct modes of action between these two closely related Ca^2+^ sensors.

The interplay between NO and Ca^2+^ constitutes a bidirectional regulatory loop that is central to plant signaling. Ca^2+^ serve as critical downstream signaling components in NO-mediated plant pathways, with their interplay exhibiting complex, context-dependent regulation. NO influences cytosolic Ca^2+^ ([Ca^2+^]cyt) dynamics through multiple mechanisms: it triggers intracellular Ca^2+^ release and modulates plasma membrane channels while altering membrane potential (Vandelle et al., 2006), yet paradoxically can also reduce [Ca^2+^]cyt by stimulating Ca^2+^-ATPases (Courtois et al., 2008). Experimental evidence demonstrates NO-induced Ca^2+^ elevation in guard cells and intracellular stores (Garcia-Mata et al., 2003), with similar regulation observed during ABA-induced stomatal closure, auxin-mediated root formation (Desikan et al., 2002), and stress responses (Vandelle et al., 2006). However, antagonistic effects exist—NO-generated cGMP activates Ca^2+^/calmodulin-dependent kinases that inhibit Ca^2+^ pumps, promoting efflux (Chai et al., 2011). Our data corroborate this duality: CaCl_2_ rescued NO-induced hypocotyl inhibition in Col-0, whereas application of SNP combined with EGTA or RR suppressed hypocotyl elongation in both Col-0 and *cml38*. Notably, however, *cml38* mutants exhibited a similar trend to Col-0 in NO-triggered [Ca^2+^]cyt increases, suggesting that CML38 acts independently of initial calcium flux. Electrophoretic mobility assays confirmed that CML38 retains Ca^2+^-binding activity unaffected by NO, indicating that NO may regulate CML38 through mechanisms beyond calcium binding. This stands in sharp contrast to CML23, whose Ca²^+^-binding ability is directly modulated by NO via *S*-nitrosylation and is indispensable for mediating the NO-induced initial cytosolic Ca²^+^ elevation (Wang et al., 2025), fully revealing the distinct mechanisms by which the two CMLs participate in NO- Ca^2+^ crosstalk.

Furthermore, compared to CML23, CML38 governs a smaller, more distinct subset of target genes. Within the Ca^2+^ signaling network, CML38 deficiency impairs NO-mediated regulation of three critical Ca^2+^-related genes: *NDB4*, which modulates mitochondrial electron transport in a Ca^2+^-dependent manner (Geisler et al., 2007); *CPK15*, as a calcium-dependent protein kinase involved in in plant growth and stress responses (Manimaran et al., 2015); *OSCA2.1*, a plasma membrane-localized Ca^2+^-permeable channel that mediates Ca^2+^ influx in response to osmotic stress (Pei et al., 2024). In light signaling, CML38 deficiency reduces *SPA1* and *GA3OX1* expression upon NO treatment, implying a potential NO–CML38–COP1/SPA regulatory axis in hypocotyl elongation. Light critically regulates hypocotyl elongation through distinct photoreceptors: phytochrome B (phyB) perceives red light and phytochrome A (phyA) mediates far-red light responses. These photoreceptors converge on the central phy-PIF-COP1/SPA pathway to modulate hypocotyl growth by controlling the stability and activity of PIF transcription factors. PIFs promote cell elongation by activating genes associated with cell expansion and gibberellin biosynthesis. Notably, phyB inhibits while phyA promotes PIF accumulation. In darkness or short days, PIFs accumulate and enhance elongation; under prolonged red light, PIFs are phosphorylated and degraded, inhibiting growth (Chen et al., 2023). The COP1/SPA complex serves as a key regulatory switch: under dark conditions, it targets photomorphogenesis-promoting proteins such as HY5 for degradation, thereby stabilizing PIFs (Ling et al., 2017). In the light, activated phytochromes suppress COP1/SPA activity, leading to HY5 accumulation and PIF degradation, which collectively restrain hypocotyl elongation (Choi and Oh, 2016).

Furthermore, CML38 mediates extensive crosstalk between NO and phytohormone signaling. CML38 deficiency alters zeatin, α-linolenic acid, inositol phosphate, and glycerophospholipid metabolism, suggesting broad effects on phytohormone biosynthesis. Additionally, CML38 deficiency upregulates *PRR3* while downregulating *PAP1*, *SPA1*, *PRR9*, and *CDF1* in the circadian rhythm pathway, linking photoperiod sensing to hormone-mediated growth regulation. The MAPK signaling pathway, which transduces multiple hormone signals, is also affected—CML38 deficiency downregulates *CAT2*, *VSP2*, and *HAI1*, impacting abscisic acid and jasmonic acid signaling. Meanwhile, ubiquitin-mediated proteolysis promotes auxin and gibberellin signaling by degrading inhibitors (e.g., AUX/IAA and DELLA proteins) (Santner and Estelle, 2010), with *UBQ8* upregulated in CML38-deficient plants.

While our transcriptomic and phenotypic data strongly suggest that CML38 operates at the intersection of NO, light, and hormone signaling, a key limitation of this study is that we have not yet definitively established the genetic hierarchy between these pathways. As rightly noted, future research should employ genetic epistasis analysis by characterizing NO sensitivity in higher-order mutants between *cml38* and key light signaling mutants (e.g., *phyB*, *pif3*, *cop1*) or hormone biosynthesis/signaling mutants (e.g., *ga3ox1*, *spa1*, *abf3*). Furthermore, utilizing hormone biosynthetic and signaling reporters or ELISA techniques in the *cml38* background under NO treatment will be essential to visually quantify and confirm the effect of CML38 on hormonal dynamics (Ondzighi-Assoume et al., 2016). These rigorous genetic and cell biological approaches will be the focus of our future work to precisely map the position of CML38 within this complex regulatory network.

## Data Availability

The datasets presented in this study can be found in online repositories. The names of the repository/repositories and accession number(s) can be found below: https://www.ncbi.nlm.nih.gov/, PRJNA1255309.

## References

[B1] Bender K. W. Rosenbaum D. M. Vanderbeld B. Ubaid M. Snedden W. A. (2013). The Arabidopsis calmodulin-like protein, CML39, functions during early seedling establishment. Plant J. 76, 634–647. doi: 10.1111/tpj.12323, PMID: 24033804

[B2] Chai Y. Zhang D. M. Lin Y. F. (2011). Activation of cGMP-dependent protein kinase stimulates cardiac ATP-sensitive potassium channels via a ROS/calmodulin/CaMKII signaling cascade. PLoS One 6, e18191. doi: 10.1371/journal.pone.0018191, PMID: 21479273 PMC3066208

[B3] Chen H. Wang W. Chen X. Niu Y. Qi Y. Yu Z.. (2023). PIFs interact with SWI2/SNF2-related 1 complex subunit 6 to regulate H2A.Z deposition and photomorphogenesis in Arabidopsis. J. Genet. Genomics 50, 983–992. doi: 10.1016/j.jgg.2023.04.008, PMID: 37120038

[B4] Choi H. Oh E. (2016). PIF4 integrates multiple environmental and hormonal signals for plant growth regulation in Arabidopsis. Mol. Cells 39, 587–593. doi: 10.14348/molcells.2016.0126, PMID: 27432188 PMC4990750

[B5] Courtois C. Besson A. Dahan J. Bourque S. Dobrowolska G. Pugin A.. (2008). Nitric oxide signaling in plants: Interplays with Ca^2+^ and protein kinases. J. Exp. Bot. 59, 155–163. doi: 10.1093/jxb/erm197, PMID: 18212029

[B6] Delk N. A. Johnson K. A. Chowdhury N. I. Braam J. (2005). CML24, regulated in expression by diverse stimuli, encodes a potential Ca^2+^ sensor that functions in responses to abscisic acid, daylength, and ion stress. Plant Physiol. 139, 240–253. doi: 10.1104/pp.105.062612, PMID: 16113225 PMC1203374

[B7] Desikan R. Griffiths R. Hancock J. Neill S. (2002). A new role for an old enzyme: Nitrate reductase-mediated nitric oxide generation is required for abscisic acid-induced stomatal closure in *Arabidopsis thaliana* . Proc. Natl. Acad. Sci. U.S.A. 99, 16314–16318. doi: 10.1073/pnas.252461999, PMID: 12446847 PMC138608

[B8] Ding X. Zhang L. Hao Y. Xiao S. Wu Z. Chen W.. (2018). Genome-wide identification and expression analyses of the calmodulin and calmodulin-like proteins reveal their involvement in stress response and fruit ripening in papaya. Postharvest Biol. Technol. 143, 13–27. doi: 10.1016/j.postharvbio.2018.04.010

[B9] Field S. Conner W. C. Roberts D. M. (2021). Arabidopsis CALMODULIN-LIKE 38 regulates hypoxia-induced autophagy of SUPPRESSOR OF GENE SILENCING 3 bodies. Front. Plant Sci. 12. doi: 10.3389/fpls.2021.722940, PMID: 34567037 PMC8456008

[B10] Garcia-Mata C. Gay R. Sokolovski S. Hills A. Lamattiina L. Blatt M. (2003). Nitric oxide regulates K^+^ and Cl^-^ channels in guard cells through a subset of abscisic acid-evoked signaling pathways. Proc. Natl. Acad. Sci. U.S.A. 100, 11116–11121. doi: 10.1073/pnas.1434381100, PMID: 12949257 PMC196936

[B11] Geisler D. A. Broselid C. Hederstedt L. Rasmusson A. G. (2007). Ca^2+^-binding and Ca^2+^-independent respiratory NADH and NADPH dehydrogenases of *Arabidopsis thaliana* . J. Biol. Chem. 282, 28455–28464. doi: 10.1074/jbc.M704674200, PMID: 17673460

[B12] Hu Z. Zhang B. Lim L. J. Y. Loh W. Z. K. Yu D. Tan B. W. Q.. (2022). S-Nitrosylation-mediated reduction of CaV1.2 surface expression and open probability underlies attenuated vasoconstriction induced by nitric oxide. Hypertension 79, 2854–2866. doi: 10.1161/HYPERTENSIONAHA.122.19103, PMID: 36263779

[B13] Jeandroz S. Lamotte O. Astier J. Rasul S. Trapet P. Besson-Bard A.. (2013). There’s more to the picture than meets the eye: Nitric oxide cross talk with Ca^2+^ signaling. Plant Physiol. 163, 459–470. doi: 10.1104/pp.113.220624, PMID: 23749853 PMC3793028

[B14] Kudla J. Batistič O. Hashimoto K. (2010). Calcium signals: the lead currency of plant information processing. Plant Cell 22, 541–563. doi: 10.1105/tpc.109.072686, PMID: 20354197 PMC2861448

[B15] Leba L. J. Cheval C. Ortiz-Martín I. Ranty B. Beuzón C. R. Galaud J.. (2012). CML9, an Arabidopsis calmodulin-like protein, contributes to plant innate immunity through a flagellin-dependent signaling pathway. Plant J. 71, 976–989. doi: 10.1111/j.1365-313X.2012.05045.x, PMID: 22563930

[B16] Ling J. J. Li J. Zhu D. Deng X. W. (2017). Noncanonical role of Arabidopsis COP1/SPA complex in repressing BIN2-mediated PIF3 phosphorylation and degradation in darkness. Proc. Natl. Acad. Sci. U.S.A. 114, 3539–3544. doi: 10.1073/pnas.1700850114, PMID: 28292892 PMC5380025

[B17] Lokdarshi A. Craig Conner W. McClintock C. Li T. Roberts D. M. (2016). Arabidopsis CML38, a calcium sensor that localizes to ribonucleoprotein complexes under hypoxia stress. Plant Physiol. 170, 1046–1059. doi: 10.1104/pp.15.01407, PMID: 26634999 PMC4734562

[B18] Lu C. H. Chen C. C. Yu C. S. Liu C. S. Liu J. J. Wei S. T.. (2022). MIB2: metal ion-binding site prediction and modeling server. Bioinformatics 38, 4428–4429. doi: 10.1093/bioinformatics/btac534, PMID: 35904542

[B19] Manimaran P. Mangrauthia S. K. Sundaram R. M. Balachandran S. M. (2015). Constitutive expression and silencing of a novel seed specific calcium dependent protein kinase gene in rice reveals its role in grain filling. J. Plant Physiol. 174, 41–48. doi: 10.1016/j.jplph.2014.09.005, PMID: 25462965

[B20] McCormack E. Braam J. (2003). Calmodulins and related potential calcium sensors of Arabidopsis. New Phytol. 159, 585–598. doi: 10.1046/j.1469-8137.2003.00845.x, PMID: 33873603

[B21] Munir S. Khan M. R. G. Song J. Munir S. Zhang Y. Ye Z.. (2016). Genome-wide identification, characterization and expression analysis of calmodulin-like (CML) proteins in tomato (*Solanum lycopersicum*). Plant Physiol. Bioch. 102, 167–179. doi: 10.1016/j.plaphy.2016.02.020, PMID: 26949025

[B22] Oh E. Zhu J. Y. Bai M. Y. Arenhart R. A. Sun Y. Wang Z. Y. (2014). Cell elongation is regulated through a central circuit of interacting transcription factors in the Arabidopsis hypocotyl. eLife 2014, e03031. doi: 10.7554/eLife.03031, PMID: 24867218 PMC4075450

[B23] Ondzighi-Assoume C. A. Chakrabort S. Harri J. M. (2016). Environmental nitrate stimulates abscisic acid accumulation in arabidopsis root tips by releasing it from inactive stores. Plant Cell 28, 729–745. doi: 10.1105/tpc.15.00946, PMID: 26887919 PMC4826012

[B24] Pei S. Tao Q. Li W. Qi G. Wang B. Wang Y.. (2024). Osmosensor-mediated control of Ca^2+^ spiking in pollen germination. Nature 629, 1118–1125. doi: 10.1038/s41586-024-07445-6, PMID: 38778102 PMC11136663

[B25] Pirayesh N. Giridhar M. Ben Khedher A. Vothknecht U. C. Chigri F. (2021). Organellar calcium signaling in plants: An update. Biochim. Biophys. Acta Mol. Cell Res. 1868, 118948. doi: 10.1016/j.bbamcr.2021.118948, PMID: 33421535

[B26] Ren H. Wang Z. Shang X. Zhang X. Ma L. Bian Y.. (2024). Involvement of GA3-oxidase in inhibitory effect of nitric oxide on primary root growth in Arabidopsis. Plant Biol. 2024, 26. doi: 10.1111/plb.13600, PMID: 38014496

[B27] Santner A. Estelle M. (2010). The ubiquitin-proteasome system regulates plant hormone signaling. Plant J. 61, 1029–1040. doi: 10.1111/j.1365-313X.2010.04112.x, PMID: 20409276 PMC3066055

[B28] Sanz L. Albertos P. Mateos I. Sánchez-Vicente I. Lechón T. Fernández-Marcos M.. (2015). Nitric oxide (NO) and phytohormones crosstalk during early plant development. J. Exp. Bot. 66, 2857–2868. doi: 10.1093/jxb/erv213, PMID: 25954048

[B29] Song X. Li J. Lyu M. Kong X. Hu S. Song Q.. (2021). CALMODULIN-LIKE-38 and PEP1 RECEPTOR 2 integrate nitrate and brassinosteroid signals to regulate root growth. Plant Physiol. 187, 1779–1794. doi: 10.1093/plphys/kiab323, PMID: 34618046 PMC8566301

[B30] Symonds K. Teresinski H. Hau B. Chiasson D. Benidickson K. Plaxton W.. (2024). Arabidopsis CML13 and CML14 have essential and overlapping roles in plant development. Plant Cell Physiol. 65, 228–242. doi: 10.1093/pcp/pcad142, PMID: 37946525

[B31] Tsai Y. C. Delk N. A. Chowdhury N. I. Braam J. (2007). Arabidopsis potential calcium sensors regulate nitric oxide levels and the transition to flowering. Plant Signal. Behav. 2, 446–454. doi: 10.4161/psb.2.6.4695, PMID: 19517005 PMC2634334

[B32] Vandelle E. Poinssot B. Wendehenne D. Bentéjac M. Pugin A. (2006). Integrated signaling network involving calcium, nitric oxide, and active oxygen species but not mitogen-activated protein kinases in BcPG1-elicited grapevine defenses. Mol. Plant Microbe In. 19, 429–440. doi: 10.1094/MPMI, PMID: 16610746

[B33] Vanderbeld B. Snedden W. A. (2007). Developmental and stimulus-induced expression patterns of Arabidopsis calmodulin-like genes CML37, CML38 and CML39. Plant Mol. Biol. 64, 683–697. doi: 10.1007/s11103-007-9189-0, PMID: 17579812

[B34] Wang D. S. Zhang X. D. Qiao Y. Y. Liu W. Z. (2025). CML23 mediates NO induced Ca2+ signaling during hypocotyl elongation in Arabidopsis. Plant Sci. 359, 112619. doi: 10.1016/j.plantsci.2025.112619, PMID: 40543816

[B35] Wang S. S. Diao W. Z. Yang X. Qiao Z. Wang M. Acharya B. R.. (2015). *Arabidopsis thaliana* CML25 mediates the Ca^2+^ regulation of K^+^ transmembrane trafficking during pollen germination and tube elongation. Plant Cell Environ. 38, 2372–2386. doi: 10.1111/pce.12559, PMID: 25923414

[B36] Wang Y. Zhang W. Z. Song L. F. Zou J. J. Su Z. Wu W. H. (2008). Transcriptome analyses show changes in gene expression to accompany pollen germination and tube growth in Arabidopsis. Plant Physiol. 148, 1201–1211. doi: 10.1104/pp.108.126375, PMID: 18775970 PMC2577266

[B37] Wu X. Qiao Z. Liu H. Acharya B. R. Li C. Zhang W. (2017). CML20, an Arabidopsis calmodulin-like protein, negatively regulates guard cell ABA signaling and drought stress tolerance. Front. Plant Sci. 8. doi: 10.3389/fpls.2017.00824, PMID: 28603528 PMC5445667

[B38] Yang X. Wang S. S. Wang M. Qiao Z. Bao C. Zhang W. (2014). *Arabidopsis thaliana* calmodulin-like protein CML24 regulates pollen tube growth by modulating the actin cytoskeleton and controlling the cytosolic Ca^2+^ concentration. Plant Mol. Biol. 86, 225–236. doi: 10.1007/s11103-014-0220-y, PMID: 25139229

