# Supplementary material for: CML38 is involved in NO-induced inhibition of hypocotyl elongation in Arabidopsis
Source: Front Plant Sci. 2025 Oct 8;16:1684245. doi: 10.3389/fpls.2025.1684245 (PMC12540314; doi:10.3389/fpls.2025.1684245)
Supplement: Supplementary file 1 [file DataSheet1.docx]

Supplementary Material

# Supplementary Figure


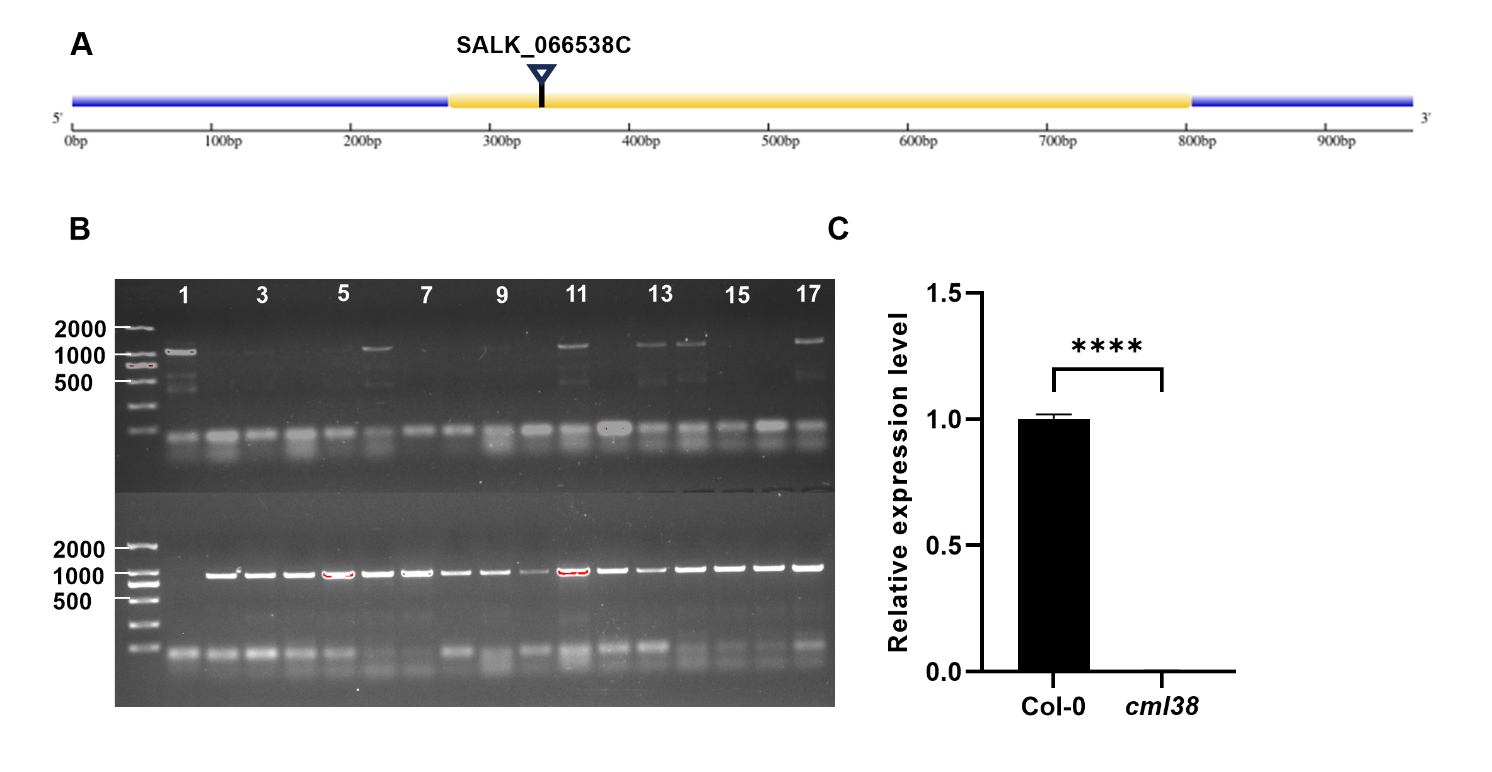


**Supplementary Figure 1.** Verification of *cml38* mutant. **(A)** T-DNA insertion site. **(B)** Identification of homozygous mutants by the three-primer method. Lane 1 corresponds to the wild type, while lanes 2 through 17 represent the identified mutants. In the upper gel, DNA amplification was carried out using primers FP and RP; in the lower gel, primers FP and LBb were employed. Samples that exhibited no amplification in the upper gel but were successfully amplified in the lower gel were identified as homozygotes. **(C)** Expression level of *CML38* in Col-0 and *cml38*.

# Supplementary Tables

**Supplementary Table S1.** Primers for RT-qPCR.

| Gene Name | Foward Primers (5’-3’) | Reverse Primers (5’-3’) |
| --- | --- | --- |
| *ACTIN2* | TTTCCCGCTCTGCTGTTGT | TGTGCCAATCTACGAGGGTTT |
| *CML38* | TCTAAACACTAATCTACCACGTCT | CTCCGGCTGAATCTTCCCTC |
| *NDB4* | TCAGCACTTTCAGTGGTGGT | TCAAGAAGCTGTAGCCGCTC |
| *CPK15* | AACTCATGGAAGCCGCTGAT | GGGACTAAACTTACCCGCTGT |
| *OSCA2.1* | TCAGAGACTGCTGCGTGATG | TCCTTTCACACTGTCTGCCTC |
| *SPA1* | TGGGATCTTCGGGGTATGGA | GGAACATCATCCACTCGTGC |
| *ARR9* | TGCGTTTTCTAAGGCTCCTGT | GCTCCTTCTTCTAAACATCTGCTG |
| *PAP1* | TTCCTGTAAGAGCTGGGCTA | GGTCCGACCAGGTAATCTTCC |
| *PER1* | TGGACCGTCCTCTTCTCTCAT | CACCTTGCTTCCGTGATTAAAG |
| *EXT4* | CTCCTCCTCCGTACACCACT | TCCCGTCAACGATCTTGTGT |
| *JAZ7* | TTCCATAGCTCGTTGGACG | ATCCGAACCGTCTGAACTTCTC |
| *PRR9* | TCCTGTCATAATGATGTCTTCTCA | GTGCTGTGAAGCTGGTAAGC |
| *ABF3* | CACTACAGATGCAGCAGCCA | TTCACTTCCTGGCACTGTGT |
| *GA3OX1* | ACATCACCTCAACTACTGCGAT | GTCTTCTTCGCTGACCCCAA |

**Supplementary Table S2.** Differential expressed genes annotated to phytohormones biosynthesis and signal transduction.

| Gene ID | Gene Name | Description | Change |
| --- | --- | --- | --- |
| Jasmonic acid |  |  |  |
| AT1G07900 | *LBD1* | LOB domain-containing protein 1 | down |
| AT1G54000 | *GLL22* | GDSL-like Lipase/Acylhydrolase superfamily protein | up |
| AT1G54040 | *ESP* | epithiospecifier protein | down |
| AT1G66100 |  | Plant thionin | down |
| AT1G70700 | *TIFY7* | TIFY domain/Divergent CCT motif family protein | down |
| AT1G76930 | *EXT4* | extensin 4 | up |
| AT2G18193 |  | P-loop containing nucleoside triphosphate hydrolases superfamily protein | up |
| AT2G22200 |  | Integrase-type DNA-binding superfamily protein | down |
| AT2G34600 | *JAZ7* | jasmonate-zim-domain protein 7 | down |
| AT2G40460 |  | Major facilitator superfamily protein | down |
| AT2G44230 |  | hypothetical protein (DUF946) | down |
| AT3G22560 |  | Acyl-CoA N-acyltransferases (NAT) superfamily protein | down |
| AT3G22740 | *HMT3* | homocysteine S-methyltransferase 3 | down |
| AT3G24420 |  | alpha/beta-Hydrolases superfamily protein | down |
| AT3G28220 |  | TRAF-like family protein | down |
| AT3G61920 |  | UvrABC system protein C | down |
| AT4G04840 | *MSRB6* | methionine sulfoxide reductase B6 | down |
| AT4G14090 |  | UDP-Glycosyltransferase superfamily protein | down |
| AT4G21840 | *MSRB8* | methionine sulfoxide reductase B8 | up |
| AT4G26850 | *VTC2* | GDP-L-galactose phosphorylase 1 | down |
| AT5G02230 |  | Haloacid dehalogenase-like hydrolase (HAD) superfamily protein | down |
| AT5G19110 |  | Eukaryotic aspartyl protease family protein | up |
| AT5G24770 | *VSP2* | vegetative storage protein 2 | down |
| AT5G35940 |  | Mannose-binding lectin superfamily protein | up |
| AT5G36907 |  | transmembrane protein | up |
| AT5G59510 | *RTFL5* | ROTUNDIFOLIA like 5 | up |
| AT3G22275 |  | jasmonate ZIM-domain protein | down |
| AT4G37150 | *MES9* | methyl esterase 9 | down |
| AT5G63450 | *CYP94B1* | cytochrome P450, family 94, subfamily B, polypeptide 1 | down |
| AT1G56650 | *PAP1* | production of anthocyanin pigment 1 | down |
| Auxin |  |  |  |
| AT1G27740 | *RSL4* | root hair defective 6-like 4 | up |
| AT4G33880 | *RSL2* | ROOT HAIR DEFECTIVE 6-LIKE 2 | up |
| AT4G37390 | *BRU6* | Auxin-responsive GH3 family protein | up |
| AT1G14185 |  | Glucose-methanol-choline (GMC) oxidoreductase family protein | down |
| AT1G20470 |  | SAUR-like auxin-responsive protein family | down |
| AT1G56650 | *PAP1* | production of anthocyanin pigment 1 | down |
| AT1G76530 |  | Auxin efflux carrier family protein | down |
| AT1G76610 |  | MIZU-KUSSEI-like protein (Protein of unknown function, DUF617) | down |
| AT2G21210 |  | SAUR-like auxin-responsive protein family | down |
| AT5G18030 |  | SAUR-like auxin-responsive protein family | down |
| AT5G64770 | *RGF9* | root meristem growth factor | down |
| AT5G65980 |  | Auxin efflux carrier family protein | down |
| AT4G30290 | *XTH19* | xyloglucan endotransglucosylase/hydrolase 19 | up |
| AT3G27025 |  | hypothetical protein | down |
| AT1G68710 |  | ATPase E1-E2 type family protein / haloacid dehalogenase-like hydrolase family protein | down |
| AT1G04180 | *YUC9* | YUCCA 9 | down |
| ABA |  |  |  |
| AT2G05100 | *LHCB2.1* | photosystem II light harvesting complex protein 2.1 | down |
| AT3G05936 |  | hypothetical protein | down |
| AT3G27690 | *LHCB2.3* | photosystem II light harvesting complex protein 2.3 | down |
| AT4G21930 |  | senescence regulator (Protein of unknown function, DUF584) | down |
| AT5G13170 | *SAG29* | senescence-associated gene 29 | down |
| AT1G24580 |  | RING/U-box superfamily protein | down |
| AT1G73390 |  | Endosomal targeting BRO1-like domain-containing protein | down |
| AT4G34000 | *ABF3* | abscisic acid responsive elements-binding factor 3 | down |
| AT4G34650 | *SQS2* | squalene synthase 2 | down |
| AT5G15500 |  | Ankyrin repeat family protein | down |
| AT5G52300 | *LTI65* | CAP160 protein | down |
| AT3G19270 | *CYP707A4* | cytochrome P450, family 707, subfamily A, polypeptide 4 | down |
| AT3G14440 | *NCED3* | nine-cis-epoxycarotenoid dioxygenase 3 | down |
| AT5G59220 | *HAI1* | PP2C protein (Clade A protein phosphatases type 2C) | down |
| Brassinosteroid |  |  |  |
| AT4G02330 | *ATPMEPCRB* | Plant invertase/pectin methylesterase inhibitor superfamily | up |
| AT5G39860 | *PRE1* | basic helix-loop-helix (bHLH) DNA-binding family protein | down |
| Salicylic acid |  |  |  |
| AT1G06137 |  | transmembrane protein | up |
| AT1G51790 |  | Leucine-rich repeat protein kinase family protein | up |
| AT1G65610 | *KOR2* | Six-hairpin glycosidases superfamily protein | up |
| AT1G66465 |  | transmembrane protein | up |
| AT1G76930 | *EXT4* | extensin 4 | up |
| AT2G04515 |  | transmembrane protein | up |
| AT4G21840 | *MSRB8* | methionine sulfoxide reductase B8 | up |
| AT5G44575 |  | hypothetical protein | up |
| AT5G13170 | *SAG29* | senescence-associated gene 29 | down |
| AT1G18870 | *ICS2* | isochorismate synthase 2 | down |
| AT4G37150 | *MES9* | methyl esterase 9 | down |
| AT4G02520 | *GSTF2* | glutathione S-transferase PHI 2 | up |
| AT1G19630 | *CYP722A1* | cytochrome P450, family 722, subfamily A, polypeptide 1 | down |
| Cytokinin |  |  |  |
| AT1G30260 |  | galactosyltransferase family protein | down |
| AT3G57040 | *ARR9* | response regulator 9 | down |
| GA |  |  |  |
| AT1G15550 | *GA3OX1* | gibberellin 3-oxidase 1 | down |
| Ethylene |  |  |  |
| AT1G04180 | *YUC9* | YUCCA 9 | down |
| AT1G56650 | *PAP1* | production of anthocyanin pigment 1 | down |
| AT4G26200 | *ACS7* | 1-amino-cyclopropane-1-carboxylate synthase 7 | up |

**Supplementary Table S3.** KEGG pathways enriched by DEGs.

| ID | Description | pvalue | Count |
| --- | --- | --- | --- |
| ath04712 | Circadian rhythm - plant | 0.000641 | 5 |
| ath00920 | Sulfur metabolism | 0.000907 | 5 |
| ath00906 | Carotenoid biosynthesis | 0.001742 | 4 |
| ath00940 | Phenylpropanoid biosynthesis | 0.002167 | 8 |
| ath04141 | Protein processing in endoplasmic reticulum | 0.005459 | 10 |
| ath00909 | Sesquiterpenoid and triterpenoid biosynthesis | 0.008004 | 3 |
| ath00100 | Steroid biosynthesis | 0.027225 | 3 |
| ath00910 | Nitrogen metabolism | 0.042975 | 3 |
| ath00196 | Photosynthesis - antenna proteins | 0.060114 | 2 |
| ath04075 | Plant hormone signal transduction | 0.079589 | 9 |
| ath00500 | Starch and sucrose metabolism | 0.091231 | 6 |
| ath00460 | Cyanoamino acid metabolism | 0.135086 | 3 |
| ath00073 | Cutin, suberine and wax biosynthesis | 0.145809 | 2 |
| ath00270 | Cysteine and methionine metabolism | 0.189977 | 4 |
| ath00900 | Terpenoid backbone biosynthesis | 0.306473 | 2 |
| ath00670 | One carbon pool by folate | 0.321335 | 1 |
| ath00904 | Diterpenoid biosynthesis | 0.321335 | 1 |
| ath00240 | Pyrimidine metabolism | 0.326753 | 2 |
| ath00380 | Tryptophan metabolism | 0.326753 | 2 |
| ath01040 | Biosynthesis of unsaturated fatty acids | 0.369753 | 1 |
| ath00630 | Glyoxylate and dicarboxylate metabolism | 0.418757 | 2 |
| ath00908 | Zeatin biosynthesis | 0.446402 | 1 |
| ath01232 | Nucleotide metabolism | 0.456303 | 2 |
| ath00770 | Pantothenate and CoA biosynthesis | 0.466555 | 1 |
| ath04016 | MAPK signaling pathway - plant | 0.468794 | 3 |
| ath00230 | Purine metabolism | 0.474559 | 2 |
| ath04146 | Peroxisome | 0.474559 | 2 |
| ath00062 | Fatty acid elongation | 0.485981 | 1 |
| ath00130 | Ubiquinone and other terpenoid-quinone biosynthesis | 0.513817 | 1 |
| ath04626 | Plant-pathogen interaction | 0.515183 | 4 |
| ath00640 | Propanoate metabolism | 0.53154 | 1 |
| ath00620 | Pyruvate metabolism | 0.532688 | 2 |
| ath00592 | alpha-Linolenic acid metabolism | 0.548623 | 1 |
| ath01200 | Carbon metabolism | 0.559536 | 5 |
| ath00480 | Glutathione metabolism | 0.565438 | 2 |
| ath00071 | Fatty acid degradation | 0.580963 | 1 |
| ath00410 | beta-Alanine metabolism | 0.580963 | 1 |
| ath00040 | Pentose and glucuronate interconversions | 0.586355 | 2 |
| ath00250 | Alanine, aspartate and glutamate metabolism | 0.611008 | 1 |
| ath03018 | RNA degradation | 0.61634 | 2 |
| ath01240 | Biosynthesis of cofactors | 0.631942 | 4 |
| ath03060 | Protein export | 0.632135 | 1 |
| ath00052 | Galactose metabolism | 0.652125 | 1 |
| ath00020 | Citrate cycle (TCA cycle) | 0.688938 | 1 |
| ath00053 | Ascorbate and aldarate metabolism | 0.688938 | 1 |
| ath00999 | Biosynthesis of various plant secondary metabolites | 0.694687 | 1 |
| ath00520 | Amino sugar and nucleotide sugar metabolism | 0.704364 | 2 |
| ath00710 | Carbon fixation in photosynthetic organisms | 0.721892 | 1 |
| ath00260 | Glycine, serine and threonine metabolism | 0.727038 | 1 |
| ath01212 | Fatty acid metabolism | 0.727038 | 1 |
| ath00562 | Inositol phosphate metabolism | 0.769311 | 1 |
| ath00564 | Glycerophospholipid metabolism | 0.841452 | 1 |
| ath00010 | Glycolysis / Gluconeogenesis | 0.891198 | 1 |
| ath03015 | mRNA surveillance pathway | 0.891198 | 1 |
| ath01230 | Biosynthesis of amino acids | 0.942407 | 2 |
| ath04120 | Ubiquitin mediated proteolysis | 0.944968 | 1 |
| ath04144 | Endocytosis | 0.948019 | 1 |
| ath03040 | Spliceosome | 0.97283 | 1 |
| ath03010 | Ribosome | 0.998992 | 1 |
