# Supplementary figures and images for: CML38 is involved in NO-induced inhibition of hypocotyl elongation in Arabidopsis
Source: Front Plant Sci. 2025 Oct 8;16:1684245. doi: 10.3389/fpls.2025.1684245 (PMC12540314; doi:10.3389/fpls.2025.1684245)

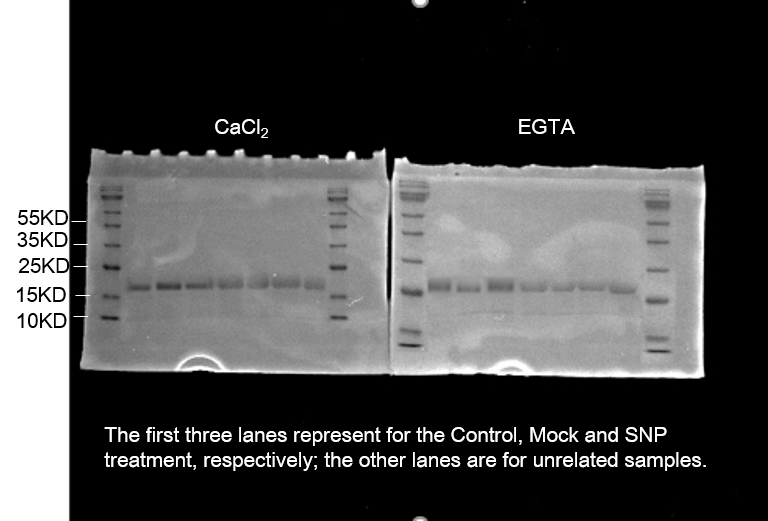

Supplement: Supplementary file 2 [file Image1.tif]

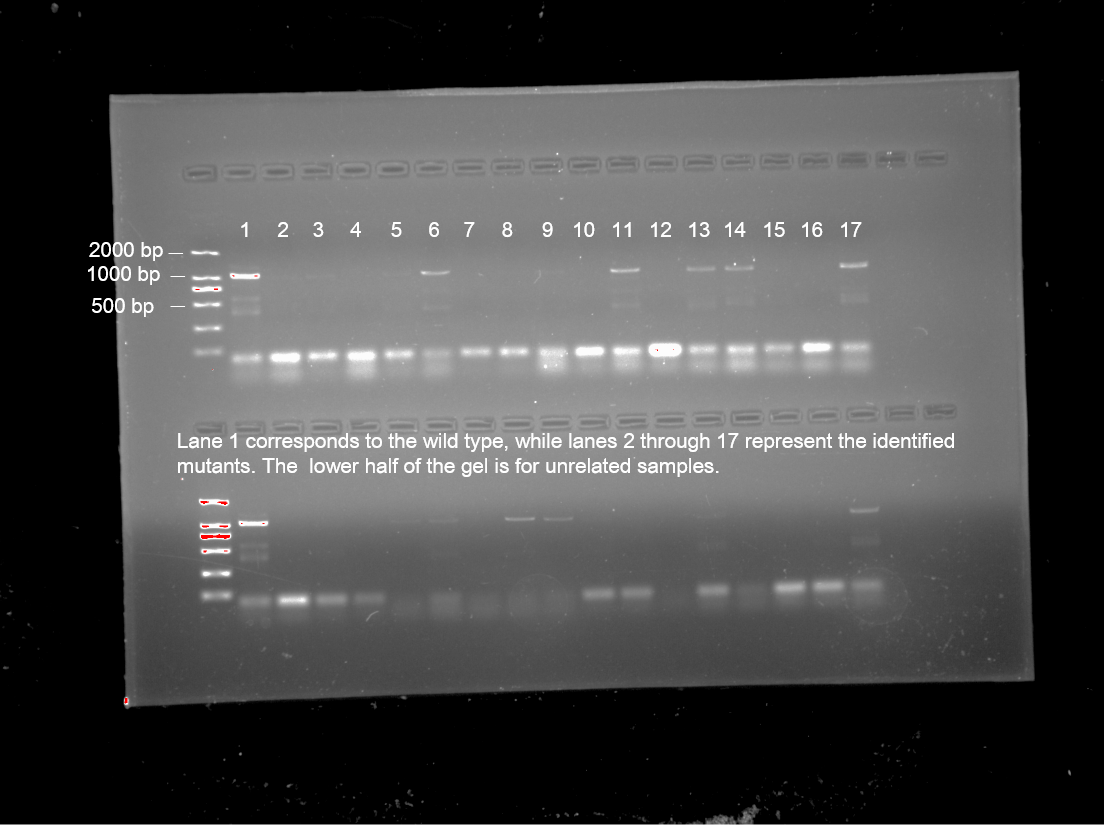

Supplement: Supplementary file 3 [file Image2.tif]

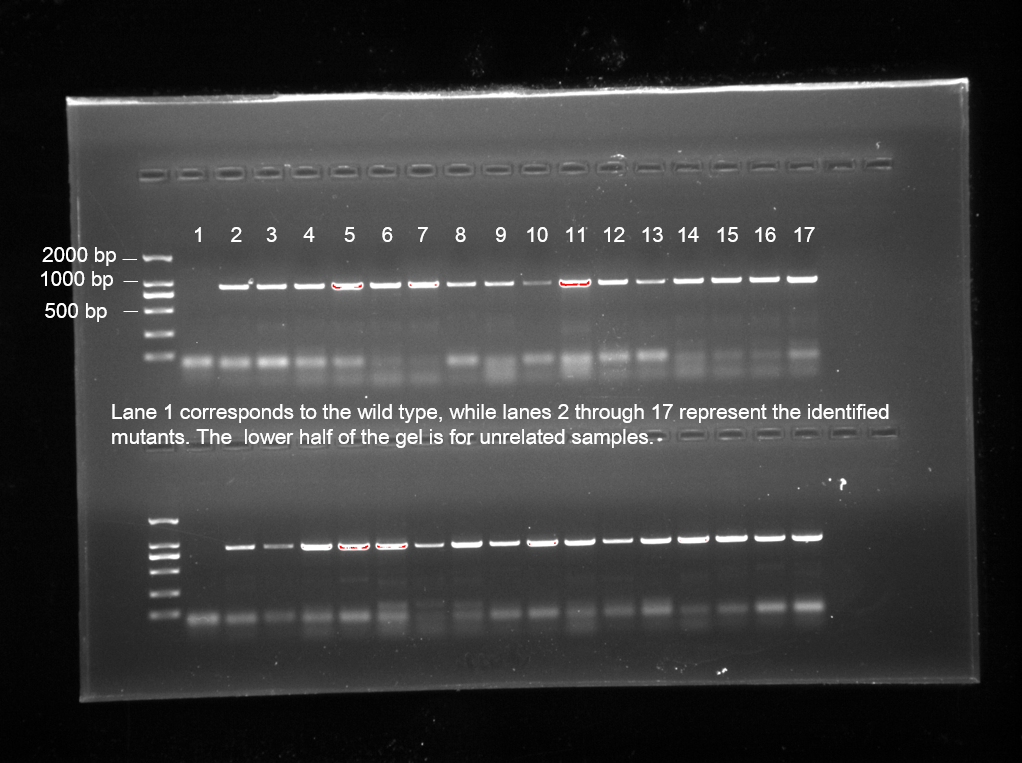

Supplement: Supplementary file 4 [file Image3.tif]
